# Rapid Growth of the World Population and Its Socioeconomic Results

**DOI:** 10.1155/2022/8110229

**Published:** 2022-03-23

**Authors:** Rahim Sadigov

**Affiliations:** Baku Business University, 88a H. Zardabi St., Baku AZ1122, Azerbaijan

## Abstract

This article is mainly devoted to the study of socioeconomic opportunities and problems that may arise from the growth of the world's population. The article identifies the reasons for the increase in world population and analyzes the factors influencing on the process. The article examines the impact of changes on the world's demographics on socioeconomic development. As a result, the characteristics of possible problems were investigated and evaluated. The study analyzes the issues of demographic change in the world population, the current situation, and opportunities of the world economy in accordance with population statistics and its growth rate. The main purpose of the study is to determine the causes of world population growth, analyze the current demographic situation, and determine and assess the forecast of future growth dynamics. The study discusses, analyzes, and evaluates the problems that can be caused by the growth of the world's population. The main problem we raise in the study is the mismatch between the rapid growth of the world's population and the socioeconomic security of the people. That is, if the issue of socioeconomic security is not resolved, the growth of the world's population would be a global social problem.

## 1. Introduction

The history of mankind covers a period of more than 3 million years. During this time, more than 80 billion people came to Earth, and today 10 percent of them live. According to statistics [1], the world's population is more than 7 billion 884 million people.

At present, the dynamics of world population growth [2] is very high. Such a rapid growth rate is already worrying humanity. Not only is the population growth in the world rapid, but this growth far exceeds the level of production and improvement of people's social needs. The content of the article is mainly related to the study of the future disproportion between population growth and the socioeconomic security of the people, as well as the problems it will cause because failure of meeting the socioeconomic needs of the population at the required level in the future will create certain problems in their lifestyle and social life. We can even note that the degree of social problems will be different in different regions.

In the article, we have classified the negative factors by regions into 2 groups. We grouped them into natural, economic, and social factors. These factors include shortages of drinkable water, deforestation, soil corrosion, declining arable land, GDP production, food shortages, wars, and diseases. If the existence or development of these factors takes place in the context of rapid population growth, then it will pose great threat to human's life and health. We conclude that most of these problems will be more prevalent, mainly in Africa and Asia.

More than 7.884 billion people live in five of the six continents in the world [1]. The world's population growth rate has been higher in the last two centuries. In the history of mankind, the world's population reached 1 billion in 1800. The second billion was reached in 130 years (1930), the third billion in 1960, that is, in 30 years, the fourth billion in 1974, the fifth billion in 1987, the sixth billion in 1999, and the last seventh billion in 2011, that is, in only 12 years. In the twentieth century, the world's population rose from 1.65 billion to 6 billion.

Although the world's population has begun to grow rapidly in the last two centuries, it has grown more in the twentieth century. Opinions about the consequences of rapid population growth vary widely in the world community. This quote from an article on the principle of population [3] expresses an important point of Malthus: The population is growing geometrically, but food production is growing arithmetically. According to Ermisch (1983), population growth can create unemployment, as well as increase conflict and crime in society. Imam [4] claimed that the birth rate in Japan was low, arguing that this decline had a negative impact on various areas of life in Japan, especially the economic sector.

In this article, we explore the methodology of the issue and assess the challenges that humanity will face terms of population growth and its future consequences. These problems can eventually lead to certain difficulties in meeting people's material and spiritual needs, as well as living standards. Meeting human material and spiritual needs is related to nature, economy, education, health, and social factors. Of course, the dynamic changes in the population must be accompanied by changes in these areas.

## 2. Purposes

The purpose of the article is to study the rate of population growth in the regions and to identify future socioeconomic problems.

## 3. Results and Discussions

The article notes that more than 7.884 billion people live on five of the world's six continents. About 77% of the world's population lives on two continents: Asia (60%) and Africa (17%). The remaining 23% settled on 3 continents. However, in Antarctica, there is no symbol of life due to severe low temperatures.  Table 1 shows the world's population by regions since 1800.

As can be seen from Table 1, the world's population grew steadily from 1800 to 2020 and now stands at 7.794 billion.

**Figure 1:**
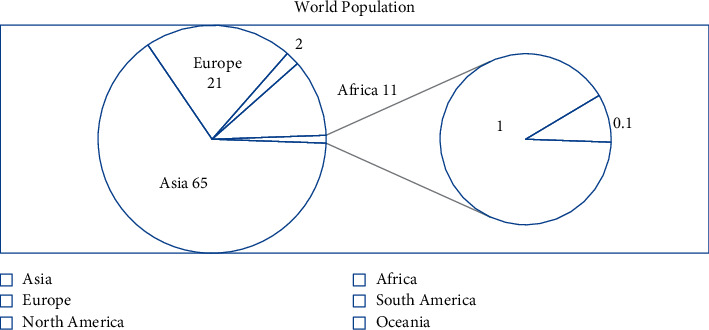
Population by regions in 1800 (source: the diagram was prepared by the author on the basis of Table 1).

If we look at Figure 1, we see that in 1800, more than half of the world's population lived in Asia. Particularly, 65% of the population lives in Asia, 21% in Europe, 11% in Africa, and the remaining 3% in North America, South America, and Oceania. However, a very small part of the world's population lived on the continents of America and Oceania. This was due to the fact that at that time the migration of people to these regions was still weak. But mass migration continued to those regions from Europe and Asia.

The population increased 52.7 times in North America, 27.2 times in South America, 21.5 times in Oceania, 12.5 times in Africa, 7.3 times in Asia, and 3.7 times in Europe. If in 1800 the population of North America, South America and Oceania was 3% of the world's population, in 2020 this figure would increase 4.7 times to 14%.

Migration from other continents has played a role in population growth in North and South America. Thus, after the discovery of America as a continent in the 15th century, there was a mass influx of Europeans in the 17th century. This influx has led to an increase in the population of the American continent.

As it can be seen from Table 1, the highest regional growth rates since 1900 have been recorded in Africa. During this period, the number of people in Africa has increased more than 10 times. At present, in countries where statistical analysis is considered a demographic explosion [6], the average number of children per woman is 3–4, while in African countries this figure reaches 5.

However, in many Eastern European and CIS countries, population growth has slowed and even declined in recent years.

Sociologists have determined that man, as a biological being, can live 100–140 years. However, in fact, as a result of various socioeconomic and environmental factors, a person lives less than this age. Recently, there is a tendency to prolong human life. According to world statistics, the average life expectancy in 1950 was 50 years, but in 2004 it increased to 66.7 years.

## 4. Forecast of World Population Growth by Regions

Recent changes in the world's population and the socioeconomic problems they will create in society require research and analysis in this area [1] According to a number of forecast sources, the world's population will exceed 11 billion by 2100 [2].

**Figure 2:**
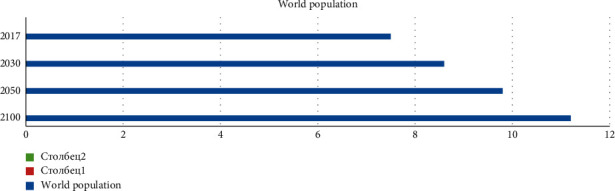
Dynamics of world's population growth forecast for 2100 by regions (source: the diagram was prepared by the author, on the basis of Table 2) (figures in million people).

According to the forecast, the world's population will exceed 8.5 billion in 2030, 9.7 billion in 2050, and 11.1 billion by 2100. As it can be seen from Table 2, the population growth rate in Africa is expected to be much higher. The continent with the highest growth dynamics in the last century was Africa. With half of the population under the age of 18, Africa's population is expected to increase by more than 3 billion by 2100.  Figure 2 shows the dynamics of world population growth by regions.

According to the forecast, if currently more than 60 percent of the world's population lives in Asia and 11 percent in Africa, the number of people living on both continents will be approximately equal during the forecast period.

One of the factors proving that the world's population growth rate will be high in the near future is that about half of the world's population is now under 25 years old. More than 3.2 billion people in the world are under the age of 15. In other words, the large number of people under the age of adolescence means that the population will grow rapidly in the near future. However, the current high growth rate of the world's population gives us reason to say that the population will increase even more for the XXI century. That is, we assume population growth as follows: taking into account the current dynamics of world population growth and applying the method of extrapolation forecasting, if the material and social needs of the growing population of the world are met at the required level.

In the forecast, the growth dynamics of the last three billion (5, 6, and 7 billion) was recorded at the frequencies of 13 and 12 years. If the dynamics of growth is estimated at this rate, the world's population could reach 14 billion by 2095. Our forecast for world's population growth by 2100 is about $3 billion higher than that of the United Nations, the Department of Economic and Social Affairs.

**Figure 3:**
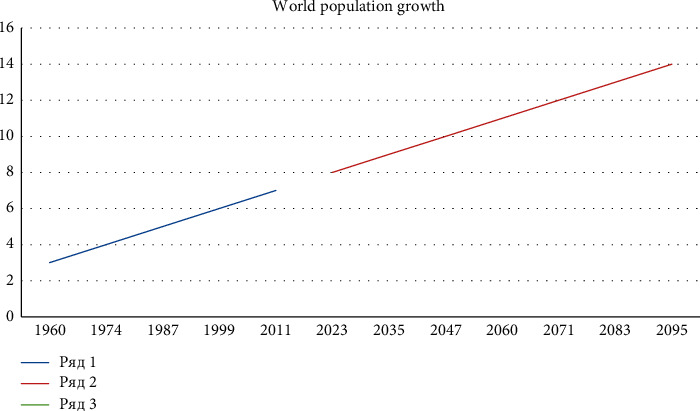
World's population growth dynamics until 2095 (the figures in the horizontal direction show the years) (Source: the table is based on two sources: author's application of mathematical-statistical extrapolation method [1] and https://en.wikipedia.org/wiki/World_population#cite_note-UN-115) (figures in billion people).

We applied the extrapolation method with a graphical model in Figure 3. Now, let us apply the extrapolation method of forecasting the world's population for 2100 with a trend model. In Table 3, we list the world's population for the last five years.

According to the trend model of the extrapolation method, the forecast for a certain period is determined by the following formula.

(1) $$\begin{array}{c} y=A_{0}+A_{1}=\frac{\sum y}{n}+t\frac{\sum yt}{\sum t{}^{*}t}. \end{array}$$

Formula (1). Trend model of the extrapolation method.

(Source of formula (1): [9]) As shown in Table 3,

(2) $$\begin{array}{c} \sum y=38151238853,\\ \sum yt=827162555,\\ \sum \left(t{}^{*}t\right)=10, \end{array}$$

*n* = 5 years (from 2016 to 2020). *t* = 85 years (from 2016 to 2100).

According to the Trend model, for the forecast period, that is, in 2100, the world's population will exceed 14.743 billion people.

(3) $$\begin{array}{c} Y_{2100}=7630247771+82716255,5{}^{*}86=14.743845744\text{ people}. \end{array}$$

Such a growth rate of the world's population must be consistent with the growth of certain macroeconomic factors. Otherwise, the world's population will face a number of socioeconomic problems, which we will discuss in the following section.

## 5. Global Problems That Society Will Face as Its Population Grows

The rapid growth of the world's population creates tasks and responsibilities for mankind. Because as a result of this growth, people may face many economic and social problems in the future. Overcoming these problems, as well as providing the resources necessary for human life, is one of the most important issues facing humanity.

**Figure 4:**
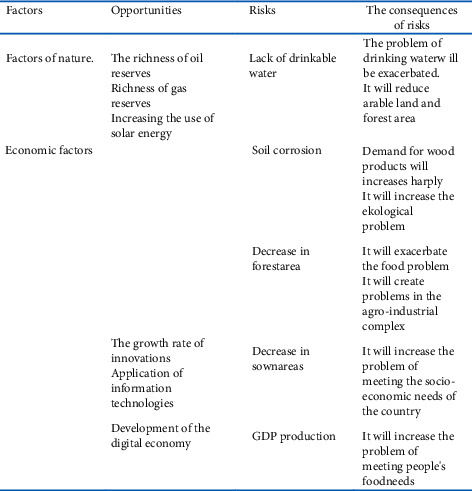
Matrix of factors influencing population growth. Figure is prepared by the author.

In our study, the problems that may occur as a result of population growth and the reasons for them are reflected in the following matrix. This matrix is illustrated in Figure 4.

The study of such factors, the analysis of the current situation, the assessment of relevant opportunities, and the assessment of their impact on the future level of social life form the methodological basis of our research. Based on the research, we have analyzed these factors as follows.

One of the biggest natural problems in the world is the problem of “drinking water.” Currently, 771 million people in the world, or one in nine people, do not have access to safe drinking water (Water Is the Way, https://water.org). At least 2 billion people worldwide use a source of water contaminated with feces. By 2025, half of the world's population will live in water-scarce areas.

Assuming that the least developed countries are located in Africa, Asia, and Latin America, 22% of these countries do not have water services, 21% do not have sewerage services, and 22% do not have waste management services. Even in Asia and Latin America, where population growth is expected to increase over the next 80 years, the problem of drinking water will worsen. However, in Africa, where the population is expected to increase by 3.128 billion people, or 3.3 times over that period, it is expected to have serious consequences in this area.

The next natural problem is the rapid deforestation. The reduction of forest areas in the world will have a negative impact on climate change, as well as a shortage of timber as a natural national resource. This will create serious problems in meeting people's demand for wood products.  Table 4 shows the change in the forest area of the regions over the years. This allows us to observe how much the forest area in different regions increased and decreased in 1900–2015.

**Figure 5:**
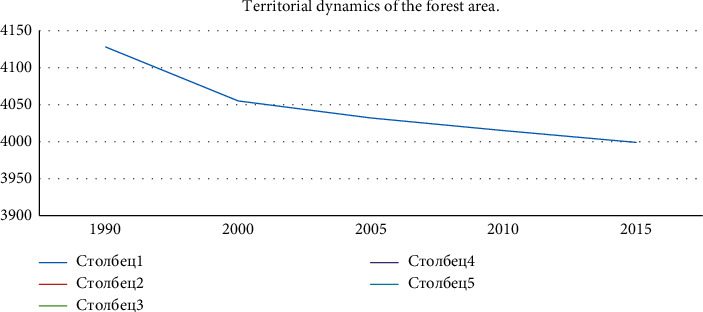
Territorial dynamics of the forest area (source: the figure was prepared by the author on the basis of Table 4).

As can be seen from Table 4, the regions with the largest forest area in the world are Europe and the Americas. The world's forest area has decreased by 129,1 million hectares over 25 years. This means that over 25 years, the world's forest area has decreased by 3.1%. As can be seen from Figure 5, the rapid dynamics of decline was observed in 1990–2000. The figures in the horizontal direction show the years, and the figures in the vertical direction show the forest area in billion hectares.

The decline is mainly in Africa and Latin America. Between 1990 and 2015, forest area decreased by 81.6 million hectares in Africa, 88.8 million hectares in South America, 1.8 million hectares in North America, and 3.3 million hectares in Oceania. If the rate of deforestation continues as in the last five years, by 2100, up to 8% of the world's forest cover will be destroyed. However, 21.2 million hectares of forests have increased in Europe and 5.1 million in Asia.

By 2100, we forecast that the population of Africa will increase by 255.7%, and forest cover will decrease by 39% (241.1 million hectares). If currently there are 496.9 thousand hectares of forest per capita in Africa, in 2100 this figure will be 85.7 thousand hectares. In other words, per capita forest area in Africa will decrease by 6.8 times by the end of the century. The sharp decline will have a negative impact on Africa's climate, forestry, wood processing, and the food industry, which is one of the most important sectors for human life. So, we can conclude from the above that the changes that may occur in the forest sector may cause serious problems for Africa, Latin America, and Oceania in the near future.

Soil erosion is also among the most likely natural problems. Soil erosion has been around for millennia as part of geological processes and climate change. Globally, corroded land covers 200,000 hectares of arable land and pastures. It covers an area larger than the United States and Mexico. More than 55% of this problem is caused by water erosion and about 33% by wind erosion.

Soil erosion affects the reduction of arable land. Every year, soil erosion and other soil degradation destroy 5–7 million hectares of arable land around the world. Every year, 25,000 million tons of topsoil are washed away. China's Huang River alone discharges 1,600 million tons of soil into the sea each year. The United States has lost about a third of its topsoil since agriculture began. So far, soil erosion has endangered the lives of about 1 billion people worldwide.

The abovementioned problems will lead to a reduction in the area under agricultural crops. This, in turn, will create significant restrictions on the production of agricultural products for the world's population. By 2100, the number of people whose lives will be at risk as a result of this problem will exceed 3 billion.

The next economic problem that we consider to be for the world's population, which is expected to increase, is the production of GDP. If there is a mismatch between world's population growth and GDP production, this problem can have serious socioeconomic consequences in those regions. Some experts do not consider the increase in the number of people to be a problem. According to Simon [11], “the benefit of population growth is the increase in the supply of intelligence and knowledge. In his view, the mind is as economically important as the hands or mouth.”

But production consists of 3 main resources.

1. Fixed assets (equipment, machines, etc.)
2. Current assets (raw materials, materials, fuel, and energy)
3. Workforce (human resources).

Human resources produce products using other resources. Man, as a social being, has intellect, thinking, and reason. But in order to produce a product, it is necessary to have equipment and technology, as well as raw materials and material resources.

In Table 5, we systematize the GDP per capita in specific regions of the world. According to statistics, the GDP per capita in Africa is $1788,61 and in South America $4360,06. Table 2 shows population growth forecasts in the African and South American regions. In case of population growth, it is necessary to increase the GDP in those regions in order to meet their material and moral needs at the current level.

If we look at the current state of GDP and the dynamics of world population growth, we see that in 2020, the African continent will produce 2.8% of world GDP. However, if by 2100 Africa's population will increase 3.3 times and make up 40% of the world's population, then the socioeconomic situation on that continent could reach a very catastrophic level. By 2100, population growth is predicted to be 1.67 times in Oceania and 1.4 times in North America. This increase will not worsen the economic situation in Oceania, which has a GDP per capita of $37,246.7. If the balance between population growth and output growth in Latin America is broken, the socioeconomic situation in the region is expected to deteriorate.

Population growth for the forecast period will not worsen the socioeconomic situation in the Asian region. But the regions with the highest per capita GDP are North America ($65371,73) and Europe ($27990,22). If the population of North America increases by 1.4 times, we expect that the socioeconomic situation in the region will remain high. Today, the North American region produces more than 28 percent of the world's GDP, more than six times the world's GDP per capita. In Europe, which has strong economic relations, although the population is expected to decline, the region's GDP is expected to increase.

There are enough energy resources in the world. According to statistics, oil reserves exceed 1,500 billion barrels. Carbon-hydrogen energy resources are already being replaced by cheap and easily available resources in the industry, such as solar energy, hydropower, and wind energy. The current availability of energy resources and the increase in opportunities for alternative energy sources give us reason to say that no problems are expected in this area in the last 100 years.

One of the most dangerous problems predicted for the end of the century is food. The food crisis has existed in certain regions of human history. However, the worst food crisis in the world since 1974 occurred in 2007 and 2008. One of the reasons for the crisis was the high prices [13] for food products (especially wheat, rice, soybeans, and corn) on the world market. This led to an increase in the level of hunger. Despite the moderate decline in prices since 2008, the number of hungry people continued to rise in 2009. This food crisis has brought the fight against hunger to the international agenda.

Some of the problems listed above can affect the food problem. These include declining arable land, soil erosion, declining agricultural water resources, and others. We have discussed these problems above, and we have assessed the level at which they will occur.

One of the reasons for the food problem is that more than 1.3 billion tons of all food products, or 1/3, are wasted. We say this according to the World Food Program. To produce this wasted food, Russia needs as much water as the annual flow of the Volga River and as much arable land as Canada (The Top 10 Causes of Global Hunger, https://www.concernusa.org/story/causes-of-global-hunger).

## 6. Conclusions

The study discusses the problems that can be caused by the growth of the world's population, and we have come to the following conclusions.

(6.1) According to the Trend model, we have set a forecast for the world's population in 2100.
(6.2) Global negative changes in natural resources and events will create socioeconomic problems at a time when the world's population is growing rapidly.
(6.3) Population growth in the world can result in various social problems in the some regions. Social problems can manifest themselves in various social spheres of society. Population growth in Latin America over the forecast period will create problems in meeting the material and spiritual needs of the population in that region. However, rapid population growth in Africa could result in very serious socioeconomic consequences.
(6.4) Although the population of Europe is expected to decrease, socioeconomic development is predicted in the region.
(6.5) Population growth in North America and Oceania will lead to an increase labor force and, consequently, will play a positive role in economic development in the regions.

## Figures and Tables

**Table 1:** World historical and predicted populations by regions (figures in million people).

*t*	1800	1850	1900	1950	1999	2010	2020
Africa	107	111	133	221	783	1.022	1.340
Asia	635	809	947	1.402	3.700	4.164	4.641
Europe	203	276	408	547	675	738	747
Latin America	24	38	74	167	508	590	653
North America	7	26	82	172	312	345	369
Oceania	2	2	6	13	30	37	43
World	978	1.262	1.650	2.521	6.008	6.896	7.794

Source: [5], https://p2k.unkris.ac.id/IT/en/3065–2962/sevenbillion-people-alive_22650_p2k-unkris.html.

**Table 2:** Regional population growth forecast for the world by 2100 (figures in million people).

Region	2017	2020	2030	2050	2100
Africa	1.256	1.340	1.704	2.528	4.468
Asia	4.504	4.641	4.947	5.257	4.780
Europe	742	747	739	716	653
Latin America and the Caribbean	646	653	718	780	712
North America	361	369	395	435	499
Oceania	41	43	48	57	72
World's population	7.550	7.794	8.551	9.772	11.184

Source: [7], Table S.2. Total population by country and region, 1950, 2017, 2030, 2050, and 2100 (medium variant); https://population.un.org/wpp/Publications/Files/WPP2017_KeyFindings.pdf.

**Table 3:** Statistical table of world population for the last five years.

Years	World's population = *y* (by people)	*t*	*t* ^2^	*yt*
2016	7464022049	−2	4	−14928044098
2017	7547858925	−1	1	−7547858925
2018	7631091040	0	0	0
2019	7713468100	1	1	7713468100
2020	7794798739	2	4	15589597478
Total	38151238853	0	10	827162555

*Source*. The table was prepared by the author on a basis of the table in [8]; https://www.worldometers.info/world-population/world-population-by-year.

**Table 4:** Forest area by regions in the world (by 1000 hectares).

Region	1990	2000	2005	2010	2015
Africa	705740,1	670372,1	654678,9	638282,2	624102,6
Asia	568121,5	565911,6	580867,6	589405,4	593361,5
Europe	994270,9	1002301,6	1004147,0	1013572,0	1015482,5
Oceania	176825,2	177641,2	176485,3	172001,6	173523,6
North America	752498,2	748558,68	747952,43	750278,61	750652,73
South America	930813,7	890817,1	868611,4	852133,2	842010,6
World	4128270,7	4055602,2	4032742,7	4015673,0	3999133,6

Source: [10], Desk Reference, Download FRA 2015 results, https://www.fao.org/forest-resources-assessment/past-assessments/fra-%202015/en/.

**Table 5:** GDP per capita in the regions of the world.

Regions	2020 GDP (trillion dollars)	Population in 2020 (billion people)	Per capita falling GDP (in dollars)
Asia	33,095.342	4,641	7131,08
Europe	20,908.694	0,747	27990,22
North America	24,122.169	0,369	65371,73
South America	2,847.121	0,653	4360,06
Africa	2,396.739	1,340	1788,61
Oceania	1,601.607	0,043	37246,7
World	84,971.650	7,794	**10902,19**

*Source*. The table was prepared by the author on a basis of two sources 1.[12], List of continents by GDP, https://statisticstimes.com/economy/continents-by-gdp.php. 2.[5], https://p2k.unkris.ac.id/IT/en/3065–-2962/seven%20-billion-people-alive_22650_p2k-unkris.html.

## Data Availability

(1) Information of Table 1 (world historical and predicted population by regions) was obtained from the following source: World historical and predicted populations, https://p2k.unkris.ac.id/IT/en/3065-2962/seven-billion-people-alive_22650_p2k-unkris.html2. Information of Diagram 1 (population by regions in 1800) was obtained from Table 1.3). Information of Table 2 (regional population growth forecast for the world by 2100) was obtained from the following source: World Population Perspectives: 2017, Table S.2. Total population by country and region, 1950, 2017, 2030, 2050, and 2100 (medium variant) https://population.un.org/wpp/Publications/Files/WPP2017_KeyFindings.pdf 4. Information of Diagram 3 (world population growth dynamics until 2095) was obtained from the following 2 sources: (1) Author's application of mathematical-statistical extrapolation method. (2) World population, https://en.wikipedia.org/wiki/World_population#cite_note-UN-115. (5) Information of Table 3 (statistical table of world population for the last five years) was obtained from the source. The table was prepared by the author on the basis of the table: World Population by Year^ https://www.worldometers.info/world-population/world-population-by-year). (6) Information of Figure 1 (matrix of factors influencing population growth) was prepared by the author. (7) Information of Table 4 (forest area by regions in the world) was obtained from the source: Global Forest Resources Assessment 2015, Desk Reference, Download FRA 2015 results, https://www.fao.org/forest-resources-assessment/past-assessments/fra-2015/en/. (8) Information of Table 5 (GDP per capita in the regions of the world) was obtained from the following sources: (1) Statistic times, List of continents by GDP, https://statisticstimes.com/economy/continents-by-gdp.php. (2) World historical and predicted populations, https://p2k.unkris.ac.id/IT/en/3065-2962/seven-billion-people-alive_22650_p2k-unkris.html.
